# Diatom-derived extracellular polymeric substances form eco-corona and enhance stability of silver nanoparticles†

**DOI:** 10.1039/d4en00232f

**Published:** 2024-06-25

**Authors:** Rocco Gasco, Isabelle A. M. Worms, Arin Kantarciyan, Vera I. Slaveykova

**Affiliations:** a Faculty of Sciences, Department F.-A. Forel for Environmental and Aquatic Sciences, Environemntal Biogeochemistry and Ecotoxicology, University of Geneva Bvd Carl-Vogt 66 1211-Geneva Switzerland rocco.gasco@unige.ch vera.slaveykova@unige.ch

## Abstract

Silver nanoparticles (nAg) are extensively used across various fields and are frequently introduced into aquatic environments, where their behavior depends on environmental conditions. Extracellular polymeric substances (EPS) derived from aquatic organisms, such as diatoms, could play an important yet to be explored role in shaping the fate of nAg in aquatic environments. This study investigates the interactions between EPS, particularly those from the diatom *Cyclotella meneghiniana*, and citrate-coated nAg. The main objective is to understand how EPS influence the behaviours of nAg in freshwater settings, in terms of modulation of the nAg surface properties, colloidal stability and dissolution. To achieve these objectives a combination of the state-of-the-art spectroscopic and imaging techniques was employed. nAg was incubated with EPS isolated from an axenic *C. meneghiniana* culture, and their interactions were explored in a simulated freshwater environment over both short-term (0–2 h) and long-term (0–72 h) periods. The study focused on the changes in nAg, examining surface modulation, colloidal stability, dissolution, EPS adsorption on nAg, and the resulting eco-corona formation. The results indicate that EPS enhance the colloidal stability of nAg and decrease their dissolution in synthetic freshwater by adsorbing onto their surface and inducing steric repulsion between nAg particles. Visualization of the eco-corona formed by diatom EPS on nAg and its impact on aggregation processes is achieved through transmission electron microscopy. The formation of the EPS corona is attributed to the presence of diverse biopolymers within EPS, particularly proteins and polysaccharides. Fluorescence quenching studies on protein fluorophores demonstrate the formation, through hydrophobic interactions, of protein–nAg complex, further confirmed by AF4-DAD-FLD-ICP-MS. In a broader context, the results of this mechanistic study imply that diatoms, through the release of EPS, may significantly influence the destiny and possibly the bioavailability of nAg in EPS-abundant aquatic environments.

## Abstract 2

Extracellular polymeric substances from the diatom *Cyclotella meneghiniana* adsorb onto citrate-coated nAg, forming an eco-corona and reducing nanoparticle dissolution and aggregation.

## Graphical abstract

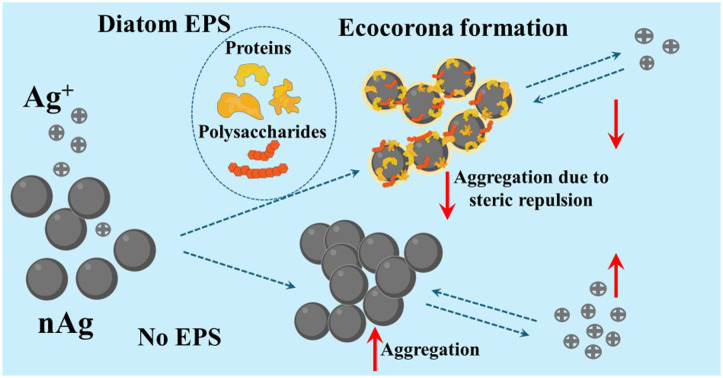

**Environmental significance**

Diatoms, through the secretion of the extracellular polymeric substances (EPS), can influence the fate of silver nanoparticles (nAg) in aquatic environments. This study highlights the significant role of diatom EPS in shaping the colloidal stability of nAg through eco-corona formation, consequently altering the fate, transport, and bioavailability of nAg in water columns. EPS constituents, particularly proteins, govern the eco-corona formation by their adsorption onto nAg and formation of complexes with nAg. The presence of an EPS corona around nAg enhances their colloidal stability by inducing steric repulsion, thereby impeding aggregation and dissolution in freshwaters. In environments where sufficient concentrations of EPS are present, eco-corona formation is likely to promote the persistence of nAg.

## 1. Introduction

Silver nanoparticle (nAg) – enabled materials are among the most used and commercialized materials due to their unique chemical and physical properties.^1,2^ Indeed, they are employed in different fields like biomedical, food and agricultural industries, mainly due to nAg remarkable antimicrobial activity.^3,4^ Their production and use are expected to increase in the future,^4^ as well as their possible release into the environment.^2,5^ Once in aquatic environments, nAg undergo physical, chemical and biological transformations,^6^ which control their fate, bioavailability, and toxicity.^7–10^ Extensive literature exists regarding the potential adverse effects of nAg on aquatic organisms,^11–13^ including phytoplankton, particularly when nAg are found in elevated concentrations. Conversely, phytoplankton species can affect the fate of engineered nanoparticles through: (i) secretion of diverse extracellular polymeric substances (EPS) that can interact with nanoparticles; (ii) cellular transformations; (iii) cellular synthesis of nanoparticles from dissolved metals.^14^ However, research investigating the role of phytoplankton in the transformations of nAg within aquatic environments remains rather scarce.

In the present study we focus specifically on the role of the phytoplankton-derived EPS on nAg behavior. The EPS produced by various aquatic microorganism are ubiquitous in the aquatic environment.^15,16^ EPS contain diverse array of macromolecules from various chemical classes including polysaccharides, proteins, lipids, nucleic acids *etc.*^17^ These substances are characterized by numerous functional groups, such as carboxyl, hydroxyl, amide, and aromatic moieties. These functional groups enable chemical interactions with metal nanoparticles, leading to the formation of a corona and intricate complexes, thereby influencing their fate and impact in the aquatic environment.^18–20^ Indeed, the adsorption of EPS on different nanoparticles, including nAg, the formation of eco-corona and the consequences for the behavior of the nanoparticles have been recently reviewed^6,19–21^ showing that the EPS released by different phytoplankton species influence nAg colloidal stability and dissolution. For example, EPS with a molecular weight greater than 1 kDa released by the cyanobacterium *Synechocystis sp.* were found to stabilize nAg with primary sizes of 20 nm and 50 nm, coated with citrate, lipoic acid, and polyvinylpyrrolidone (PVP).^22^ The stabilization effect of the EPS was significant for suspensions of citrate- and lipoic acid- coated nAg, but less pronounced for PVP-coated ones. EPS from the green algae *Chlorella vulgaris* and *Chlamydomonas reinhardtii* stabilized both PVP- and citrate-coated 20 nm-sized nAg, reduced their dissolution and efficiently complexed Ag^+^.^23^ Similarly, the EPS from other green alga *Raphidocelis subcapitata* strongly prevented the dissolution of citrate- and PVP-coated nAg.^24^ However, the composition of EPS was not consistently characterized. Alginate, an extracellular polysaccharide released by cyanobacterium *Microcystis aeruginosa*, adsorbed to 20 nm citrate-coated nAg, reducing their dissolution rate.^25^ Despite these advancements, the precise role and the underlaying mechanisms governing the interactions of the EPS released by phytoplankton species and nAg are still understudied.

The overall goal of this study is to get deeper insight on the interaction of the EPS produced by a freshwater phytoplankton with nAg. The hypothesis is that the exogenous biomolecules secreted by the phytoplankton into the aquatic environment play an important yet not fully elucidated role in surface modulation, colloidal stability and dissolution of nAg. The specific objectives are therefore: (i) to explore the role of the EPS in the colloidal stability of the nAg in terms of aggregation and dissolution; (ii) to assess the possible formation of the EPS corona and characterize the EPS–nAg complex formation. To achieve these goals, a multimethod approach combining various spectroscopic and imaging techniques was employed. The EPS produced by diatom *C. meneghiniana* were studied, as diatoms are widely present in the aquatic environments and contribute to 20% of the annual global carbon fixation.^26^ They are also known to produce significant amount of the EPS,^27^ which is rich in heterogeneous polysaccharides and proteins.^28^ In addition, the diatom-released EPS interactions with nAg are not well explored.

## 2. Material and method

### 2.1 nAg and different reagents

20 nm-sized citrate-coated silver nanoparticles (nAg) were obtained from nanoComposix (San Diego, CA, USA). In the 2 mM sodium citrate solution, the nAg had a diameter of 19.9 ± 2.8 nm, a hydrodynamic diameter of 32.93 ± 0.14 nm and a zeta potential of −39.93 ± 1.62 and a polydispersity index (PDI) of 0.265 ± 0.001 (Table S1†).

Anthrone reagent (>96.0%) was bought from Fluorochem (Hadfield, UK). Protein standards (bovine serum albumin, BSA (≥98%), equine myoglobin (≥95%), bovine hemoglobin, and horseradish peroxidase, glucose (≥99.5%), silver standard for ICP-MS were all purchased from Sigma Aldrich – Merck (Darmstadt, Germany). Ultrapure water (18.3 MΩ cm) produced with a water purification system (Milli-Q Gradient, Merck-Millipore, Darmstadt, Germany) was used throughout the experiments.

### 2.2 EPS production and characterization

To obtain the EPS, axenic culture of unicellular diatom *C. meneghiniana* (strain CCAC 0039, Central Collection of Algal Cultures, University of Duisburg-Essen, Germany) was grown in synthetic freshwater medium containing silica (SFM+Si, Table S2†).^29^ The EPS released by *C. meneghiniana* were isolated and preconcentrated from the cultures in stationary phase using an additive-free method adapted from the literature.^22,30^ Details are provided in the ESI.†

A multi-method approach was adopted to acquire diverse characteristics of the EPS. The total organic carbon (TOC) and total nitrogen (TN) contents were measured using a TOC/TN analyzed (TOC-LCPH/CPN, Shimadzu, Japan). Total polysaccharide content of the EPS was determined by anthrone–sulfuric method^31^ using glucose as standard. Total protein content was assessed by Bradford assay^32^ with BSA as standard. The zeta potential of EPS was measured by Zetasizer Nano ZS (Malvern Panalytical, Malvern, UK). Fluorophores in the EPS were characterized by three-dimensional excitation emission matrix (3D-EEM) using a fluorescence spectrophotometer (Cary Eclipse, Agilent, CA, USA). Various functional groups present in the EPS were obtained by FTIR ATR spectrometer (FTIR spectrum two+ ATR, Perkin Elmer, MA, USA). The asymmetrical flow field-flow fractions (AF4, AF2000 Focus) coupled online with diode array (DA) and fluorescence (FL) detectors (Postnova Analytics, Landsberg, Germany) were used to evaluate the molecular weight of the EPS protein with operating conditions shown in Table S3.†

### 2.3 Characterization of nAg stability and dissolution

The EPS-induced changes in the size distribution, surface properties and stability of nAg were characterized. To this end 4 mg L^−1^ nAg were mixed with increasing EPS concentrations of 10.5, 26.3, 52.5 and 105.0 mg C L^−1^ and incubated during 72 h at constant temperature of 20 °C. The EPS concentrations were chosen to cover a large concentration range, including those found in eutrophic shallow lakes.^33^ The concentration of nAg was selected for analytical purposes. Most analytical techniques for characterizing nanoparticle systems require high working concentrations, typically in the mg L^−1^ range, as documented in the literature.^18,34^

The incubations were conducted in dark, to avoid potential effect of light on nAg stability^35^ and possible formation of new nAg by EPS from dissolved Ag.^36^ Experiments in the absence of the EPS were performed as controls under the same conditions. The UV-vis absorbance spectra of nAg with a characteristics surface plasmon resonance (SPR) peak around 390 nm was recorded in the absence and presence of the EPS with microplate reader (BioTek Synergy H1 Hybrid, Bucher Biotec, Switzerland) in the range from 300 to 700 nm. The hydrodynamic diameter and zeta potential of the nAg were measured by Zetasizer Nano ZS. nAg size distributions were also characterized by transmission electron microscopy (TEM, Talos™ L120C TEM, Thermo Fisher Scientific, MA, USA) in the absence and presence of 10.5 and 105 mg C L^−1^ EPS at the incubation times of 2, 24 and 72 h as detailed in the ESI.† The dissolution of the nAg in the absence and presence of the EPS was quantified *via* centrifugation (3 h at 24 000 × *g* at 4 °C) according to the procedure reported in the literature^7^ after optimization followed by inductively coupled plasma mass spectrometry (ICP-MS) measurement (7700x Q-ICP-MS, Agilent, USA). Further details are given in ESI.†

### 2.4 Assessment of EPS corona formation on nAg

To assess the involvement of polysaccharide and proteins in the formation of EPS corona on nAg, 6 mgL^−1^ of nAg were incubated with EPS of different concentrations (Table S4†) for 2, 24 and 72 h. Next, the suspensions containing nAg + EPS were centrifuged at 25 000 × *g* at 4 °C for 30 min (5417R Refrigerated Centrifuge, Eppendorf, Hamburg, Germany). The supernatant was collected, and the concentrations of the total proteins and carbohydrates were quantified as described in the §2.1. The amount of the proteins and polysaccharides adsorbed to the nAg was determined as a difference in the measured concentrations of the polysaccharides or proteins prior and after addition of the nAg. Furthermore, the fluorescence quenching effect of nAg on protein fluorophore was studied using a spectrofluorometer (Agilent Cary Eclipse, Santa Clara, CA, USA). The detailed procedure and analysis of fluorescence quenching experiments are provided in the ESI.† The AF4 coupled online with DA, FL detectors and ICP-MS was used to evaluate the dynamics of protein corona formation on the nAg.

## 3. Results and discussion

### 3.1 Characterization of the EPS released by diatom *C. meneghiniana*

The concentrations of TOC and TN in the preconcentrated EPS isolates were 525 mg C L^−1^ and 100 mg N L^−1^, respectively. The protein fraction represented 43 mg BSA equivalent per L and the polysaccharide of 610 mg glucose equivalent per L (Table S4†). Two fluorescence peaks with at Ex/Em maxima of 255/308 nm and 280/333 nm were observed in the 3D-EEM (Fig. S1A†), corresponding to the tryptophan-like fluorophores,^16^ thus evidencing the presence of proteins and aromatic groups, that can interact with nAg through different mechanisms.^37,38^ FT IR peaks corresponding to various functional groups mainly found in the proteins, polysaccharides, lipids and phospholipids were observed in the EPS released by diatoms (Fig. S1B, Table S5†).

The present results are consistent with the existing literature showing that the diatoms' EPS are rich in polysaccharides^17^ and proteins.^39^ Two proteins with molecular weight of 35 kDa and 164 kDa, respectively, were identified by AF4-FLD (Fig. S2†). Overall, the above results demonstrated that the diatom *C. meneghiniana* release the EPS rich in polysaccharides and proteins.

### 3.2 Effect of EPS on the colloidal stability and dissolution of nAg

**Fig. 1:**
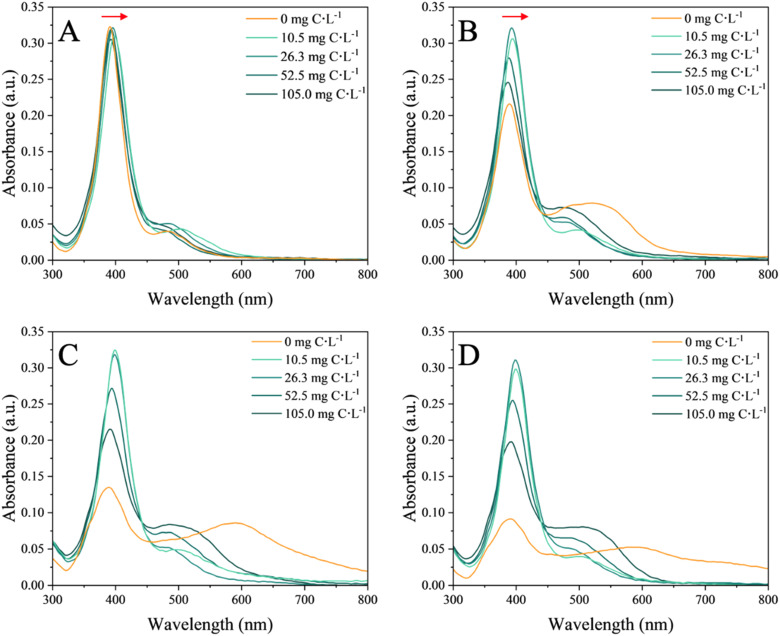
UV-vis absorbance spectra of 4 mg L^−1^ nAg in the absence and presence of increasing concentration of EPS (10.5, 26.3, 52.5, 105.0 mg C L^−1^) at the different incubation times: (A) 2 min, (B) 2 h, (C) 24 h and (D) 72 h. SPR characteristic peak of nAg is observed at 392 nm. The red arrows in (A) and (B) represent the red shifts.

The UV-vis spectra recorded in nAg suspensions in the absence and presence of the EPS provided an overview on the aggregation state and the changes of surface characteristic of the nAg (*i.e.* due to the adsorption of the EPS) (Fig. 1).

In the absence of EPS, the SPR characteristic peak of nAg centered at 392 nm rapidly decreased with the incubation time (Fig. S3H†). This decrease was accompanied by the appearance of a second peak at higher wavelength and observed already after 15 min of incubation (Fig. S3E†), suggesting a rapid aggregation process. This second peak further shifted to a higher wavelength as the incubation time increased. A similar pattern of temporal evolution was observed for the SPR peak of nAg in the presence of high concentrations of EPS. At the highest concentrations of 52.5 and 105.0 mg C L^−1^ EPS, a significant decrease of the intensity of the SPR characteristic peak of nAg (Fig. S3G†) and an increase in the intensity and the shift towards higher wavelength (545 nm in the presence of 105.0 mg C L^−1^) of the second peak were found. At the lowest tested concentrations of 10.5 and 26.3 mg C L^−1^ EPS the intensity of both UV-vis peaks remained comparable over time. Overall, a higher concentration of EPS induced stronger decrease of the SPR peak intensity and more pronounced second peak.

Based on the above observations, the hypothesis was formulated that in the absence of EPS, a significant homo-aggregation of nAg occurs, controlled by the mono- and divalent ions present in the exposure medium. These ions shield the electrostatic repulsion between the nAg leading to aggregation and increased suspension instability.^40,41^ In the presence of EPS, the EPS molecules adsorbed onto the surface of nAg, forming a corona.^42^ A red shift in the SPR nAg peak observed in presence of EPS (Fig. S3F†) suggested an increase in the local refractive index due to adsorption of EPS on the surface of nAg.^43^ This eco-corona formation mitigates the formation of large aggregates, but may enable the bridging of nAg and thus to be responsible of a more pronounced shift in the second peak, as previously observed for microbial EPS with nAg.^18^

**Fig. 2:**
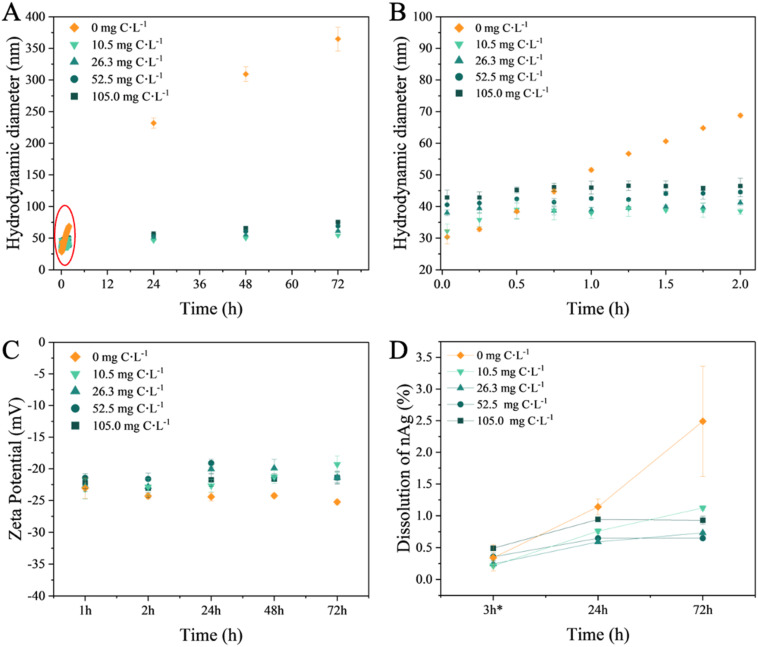
Influence of the EPS on the colloidal stability and dissolution of the nAg. (A) Time course of the hydrodynamic diameter, (*Z*-averaged) from 2 min to 72 h; (B) zoom to illustrate the time evolution of the hydrodynamic diameter during first 2 h; (C) change of the zeta potential of nAg; (D) percentage of the dissolved nAg. nAg concentration: 4 mg L^−1^, EPS concentrations: 10.5, 26.3, 52.5, 105.0 mg C L^−1^. Experiments were performed in triplicate.

The above findings were confirmed by the time evolution of the hydrodynamic diameter in nAg suspensions (Fig. 2A). In the absence of EPS, the hydrodynamic diameter increased linearly over time, reaching a value of 68.77 ± 0.68 nm at 2 h (Fig. 2B), and continued to rise to 364.64 ± 18.88 nm at 72 h of incubation, thus confirming the significant homo-aggregation in nAg suspensions. The absolute value of the negative zeta potential of nAg in the absence of EPS increased over time (Fig. 2C), which could be due to the neutralization of the surface charge by the presence of mono- and divalent ions in the medium.^44^ Such a decrease in the electrostatic repulsion which stabilizes nAg led to a subsequent rise in homo-aggregation processes likely due to Van der Waals forces.^41^ Conversely, in the presence of EPS at 2 h incubation, the hydrodynamic diameter rapidly increased reaching values between 38.50 ± 0.91 nm and 46.49 ± 2.45 nm depending on EPS concentration (Fig. 2B), demonstrating EPS concentration-dependent hetero-aggregation. An increase of the hydrodynamic diameter to 75.57 ± 1.20 nm over time was observed at 72 h (Table S6†). Alongside, the zeta potential decreased in negativity from −25.23 ± 0.38 mV in the absence of EPS to −21.40 ± 1.00 mV in the presence of 105 mg C L^−1^. These results indicate that EPS adsorbs on the nAg and mitigate the formation of the large nAg aggregates. This observation aligns with the role of EPS in bridging nAg in hetero-aggregation processes.^18^

The present results agree with existing literature demonstrating a corelation between the changes in the zeta potential of nTiO_2_ and nAg, and the adsorption of the EPS derived from *C. reinhardtii* and *C. vulgaris.*^31,34^ The adsorption of EPS increased the zeta potential to higher values while enhancing the colloidal stability of nAg. This alteration shifted the stabilizing mechanism from solely electrostatic repulsion, as seen in citrate-coated nAg, to a combined effect with steric repulsion stabilization in EPS–nAg. This is consistent with findings regarding the effects of *C. vulgaris* and *Microcystis sp.* EPS on nZnO colloidal stability.^19^ Various other biomolecules have also been demonstrated to adsorb onto nAg. For example, cytochrome *c*,^45^ antioxidant enzymes catalase and superoxide dismutase^46^ and BSA^47^ adsorbed onto nAg followed by increase of the hydrodynamic diameter. Moreover, it has been demonstrated that polysaccharides of microbial EPS adsorb on nAg and enable intermolecular bridging through calcium complexation.^18,40^

In the absence of EPS, nAg dissolution remained relatively low under the studied conditions: after 72 h, the concentration of dissolved Ag reached only 2.5% of the total Ag content in the nAg suspension.

Increasing concentrations of EPS led to a reduction in dissolution (Fig. 2D). After 72 h, the percentage of Ag ions present in the nAg suspensions was below 1.0%. The decrease in the nAg dissolution by diatom EPS aligns with findings in the literature regarding other phytoplankton species. For instance, EPS derived from green algae *Chlorella pyrenoidosa* and *Raphidocelis subcapitata* have been shown to lower the dissolution of citrate- or PVP-coated nAg.^23,24^ Nevertheless, higher EPS concentrations have been shown to lead to slightly higher dissolved Ag. Similar trend was observed for *C. vulgaris* EPS and nAg.^34^

**Fig. 3:**
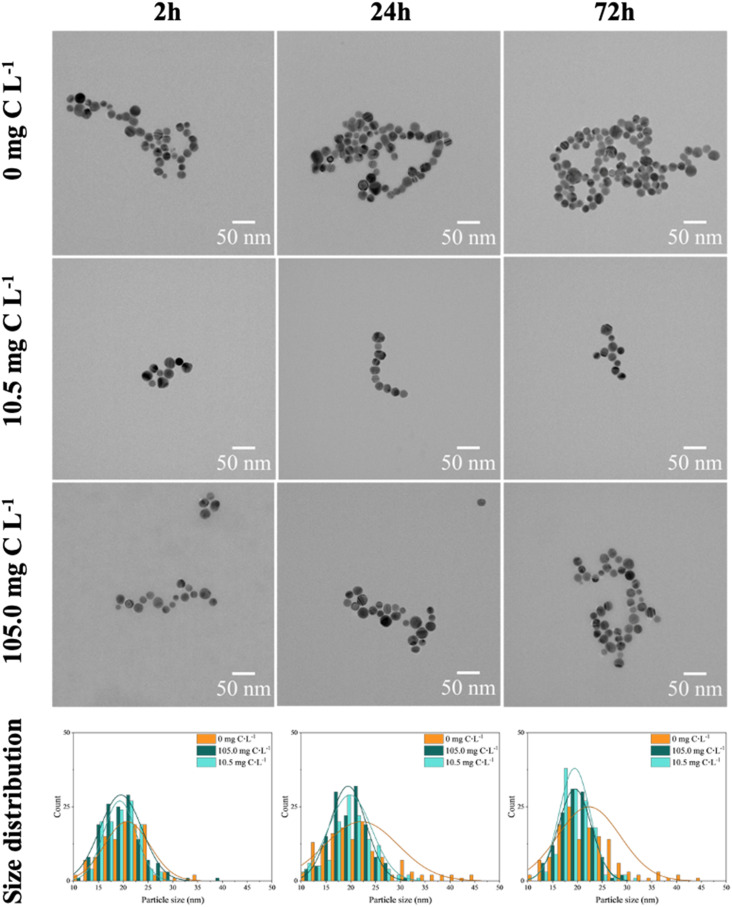
TEM images of 4 mg L^−1^ nAg in the absence and presence of EPS (10.5 and 105.0 mg C L^−1^ EPS). The different size distributions were obtained counting at least 120 individual particles.

The aforementioned observations were further supported by TEM images, which revealed the formation of larger aggregates in the absence of EPS (Fig. S4C†) and the presence of smaller hetero-aggregates in the presence of EPS (Fig. S4E and F†). Furthermore, the hetero-aggregation exhibited a dependence on EPS concentration, with larger aggregates of nAg observed at the highest EPS concentration (Fig. 3 and Table S6†). Regarding the size distribution of nAg, the formation of larger-sized particles, reaching a diameter of 45 nm, was clearly observed in the absence of EPS (Fig. S4A and S5B†). This phenomenon can be explained by Ostwald ripening.^48^ During the incubation period, smaller nAg or dissolved Ag^+^ were formed, and due to their higher surface energy, they deposited onto larger particles with lower surface energy.^49,50^ Additionally, after 24 h, the nAg formed larger aggregates, thus aligning with the increased hydrodynamic diameter measurements (Fig. S4C and D†). This phenomenon is consistent with findings for nAg homo-aggregation in various media with differing ionic strengths.^41^ In addition, the polydispersity index (PdI) values (Table S6†) related to the homo-aggregation process in the absence of EPS increased over time, indicating a more polydisperse system.

In the presence of EPS, the size distribution of nAg (Fig. 3) remained unchanged, indicating the absence of significant formation of larger nAg, due to the stabilization role of the adsorbed EPS. In addition, the nAg appeared to be more spatially separated likely due to steric repulsion resulting from the adsorbed EPS (Fig. S3B†). As a result, smaller clusters of nAg were formed by EPS bridging, and corroborated the overall trend observed in hydrodynamic diameter and zeta potential measurements (Fig. 2A). In addition, higher EPS concentration leaded to higher hetero-aggregation due to bridging role of adsorbed biomolecules.

### 3.3 Formation of EPS corona, EPS–nAg complex and underlying mechanisms

**Fig. 4:**
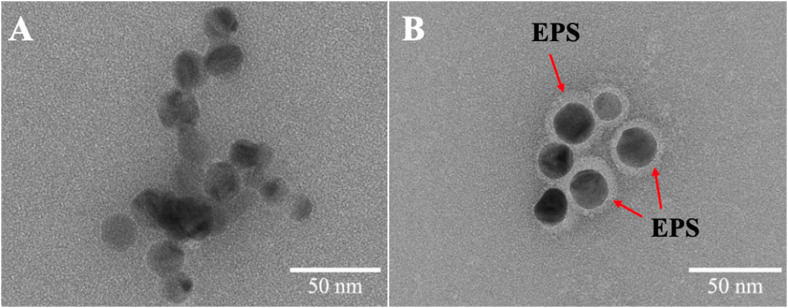
Illustration of the formation of the EPS corona on nAg. TEM images of 4 mg L^−1^ nAg in the absence (0 mg C L^−1^) (A) and presence of 105.0 mg C L^−1^ EPS (B) incubated for 2 h. Sample were stained with uranyl acetate.

The EPS adsorbed onto and formed a corona around nAg, visible in TEM images (Fig. 4B). Negative staining with uranyl acetate revealed the formation of a bright EPS corona around nAg in the presence of 105.0 mg C L^−1^ EPS, which was absent when nAg were not treated with EPS (Fig. 4A). Similar results were obtained for BSA protein corona formation on nAg.^51^ The presence of a thin amorphous EPS layer, which appeared to play an intermolecular bridging role, on the surface of nAg in presence of 10.5 and 105.0 mg C L^−1^ EPS was also observed in the unstained TEM images, and not observable in the absence of EPS (Fig. S5†). Such surface layer formation on nAg is reported in literature for protein corona formation, as for BSA and different globular proteins on nAg and nAu, respectively,^52,53^ and EPS from bacterium *Escherichia coli* on nAg.^16^

**Fig. 5:**
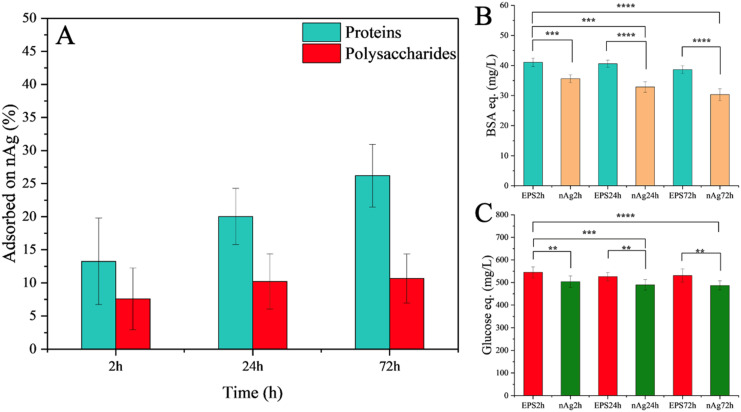
Adsorption of protein- and polysaccharide-components onto the nAg at different incubation times (2, 24 and 72 h). *EPSxh*: time related components (proteins/polysaccharides) concentration in EPS without nAg. *nAgxh*: time related components (proteins/polysaccharides) concentration in EPS incubated with nAg. (A) Adsorption of proteins and polysaccharides, quantified proteins (B) and polysaccharides (C) involved in hard corona. The asterisk represents the statistically significant difference obtained by *F*-test (ns: *p* > 0.05, *: *p* ≤ 0.05; **: *p* ≤ 0.01; ***: *p* ≤ 0.001; **** *p* ≤ 0.0001).

The percentage of the adsorbed proteins and polysaccharides on the nAg was quantified as a difference in the dissolved total proteins or polysaccharides in the EPS before and after incubation with nAg (Fig. 5). Diatom polysaccharides contribute to eco-corona formation, as indicated by the decrease in free polysaccharides in the solution after interaction with nAg. The role of exopolysaccharides in eco-corona formation is not extensively covered in the literature, being primarily noted in isolated studies on microbial exopolysaccharides and their bridging role in the hetero-aggregation process of citrate-coated nAg.^40,54^ Diatoms are recognized as one of the most prolific producers of polysaccharides among microalgae^55^ and their polysaccharides likely facilitate the formation of hetero-aggregates, as suggested by the hydrodynamic diameter results. Polysaccharides from other microalgae, such as those from the green algae Chlorella,^34^ can also interact with nAg, although these interactions are weaker than those involving proteins. A higher percentage of proteins was adsorbed to the nAg relative to their content in the EPS before incubation, compared to the relative percentage corresponding to the polysaccharides. Such higher percentage of the adsorbed proteins on nAg compared to the dissolved protein is probably due to the presence of functional group with a stronger affinity to nAg.^34^ The percentage of adsorbed proteins increased over the incubation time, while no significant change was observed in the adsorbed polysaccharides (Fig. 5).

Given the important role of the proteins in the EPS corona formation, the mechanisms of interaction between proteins and nAg were further investigated *via* fluorescence quenching study and AF4-DAD-FLD-ICP-MS.

**Fig. 6:**
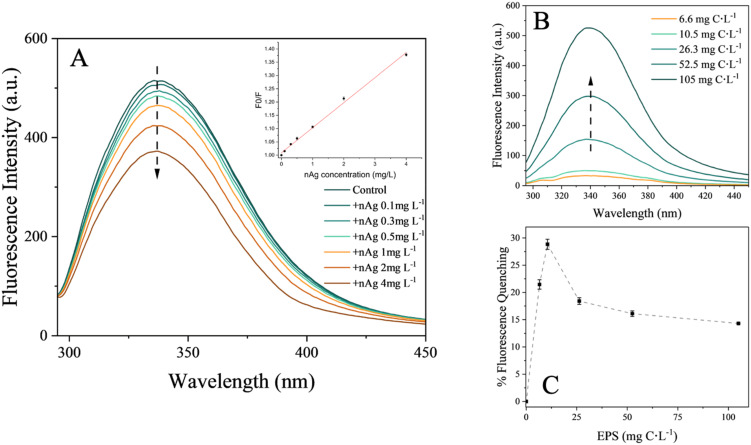
Effect of nAg on EPS fluorescence. (A) Fluorescence intensities of tryptophan-like peak (Ex: 280 nm, Em: 290–450 nm) of 26.3 mg C L^−1^ EPS in presence of increasing nAg concentrations (0.1, 0.3, 0.5, 1.0, 2.0, 4 mg C L^−1^) with the Stern–Volmer plot at 298 K derived for the binding of nAg to EPS as insert. (B) Fluorescence intensities of tryptophan-like fluorophores of the EPS at different concentrations (6.6, 10.5, 26.3, 52.5, 105.0 mg C L^−1^) in the presence 2 mg L^−1^ nAg. (C) Fluorescence quenching (%) of different EPS concentrations (6.6, 10.5, 26.3, 52.5, 105.0 mg C L^−1^) in presence of 2 mg L^−1^ nAg, derived from (B).

The fluorescence intensity related to the protein tryptophan-like fluorophores progressively decreased with increased ratios of nAg over EPS. These ratios were varied in two ways: by keeping the EPS concentrations constant while increasing the nAg concentration (Fig. 6A), and by keeping the nAg concentration constant while increasing the EPS concentrations (Fig. 6B). The results suggest at first that the proteins may be involved in the binding of nAg.^16,56^ Since the fluorescence peak was slightly red-shifted from 336 to 338 nm, it is likely that a decrease in hydrophobicity in the microenvironment around tryptophan groups occurred due to the addition of nAg.^47^ This finding is consistent with a previous study involving catalase and superoxide dismutase interacting with citrate-coated nAg.^46^ The Stern–Volmer plot at 298 K (Fig. 6A, insert) exhibited a linear relationship between quenching and the increase in nAg concentrations, suggesting that static quenching could be the dominant interaction mechanism due to the formation of a complex between the fluorescent molecule and the quencher.^57^ The increase in the % of fluorescence quenching of the EPS up to 10.5 mg C L^−1^ followed by decrease in the quenching for higher EPS concentrations reveals that there is a critical EPS concentration (nAg to EPS ratio) at which nAg are saturated under study conditions (Fig. 6C). This observation is consistent with critical concentration of BSA found for its adsorption on nAg.^47^

The obtained quenching rate constant (*K*_q_) was 1.34 × 10^12^ L mol^−1^ s^−1^, two orders of magnitude higher than the maximum *K*_q_ value for a diffusion-controlled quenching, confirming binding interaction.^58,59^ These results confirm a static quenching process due to the formation of the EPS–nAg complex. Moreover, the binding constant (*K*_b_) for EPS to nAg was higher than 10^2^ L mol^−1^ and increased with rising temperature from 298 to 308 K (Table 1). This confirms the hypothesis of EPS–nAg complex formation, with its stability being enhanced by a rise in temperature.^56,60^ The negative value of Gibbs free energy (Δ*G*°) (Table 1) showed an exothermic reaction for EPS binding to nAg. The positive values of both enthalpy (Δ*H*°) and entropy (Δ*S*°) demonstrated that the process is entropy-driven and endothermic, indicating the possible involvement of hydrophobic forces in the binding process.^61^ Similar results were found for binding of microbial EPS^16^ and lysozyme^60^ with nAg.

**Table 1:** Binding constants and thermodynamic parameters of the interactions between EPS and nAg at different temperatures (298, 303, 308 K)

*T* (K)	*K*_q_ (10^12^ L mol^−1^ s^−1^)	*K*_b_ (10^2^ L mol^−1^)	*n*	Δ*H*° (kJ mol^−1^)	Δ*S*° (J mol^−1^ K^−1^)	Δ*G*° (kJ mol^−1^)	*R* ^2^
298	1.34	2.17	0.64	67.20	270.42	−13.39	0.987
303	1.21	3.04	0.68			−14.74	0.998
318	1.34	4.27	0.71			−16.09	0.989

**Fig. 7:**
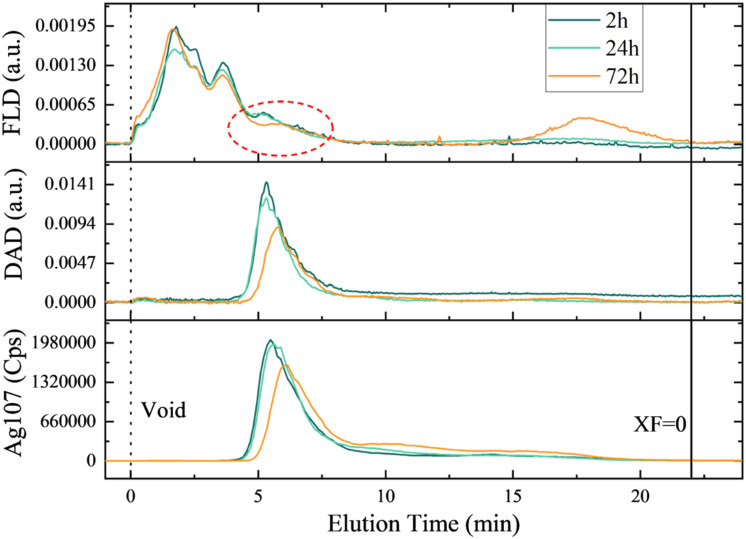
Fractograms of 4 mg L^−1^ nAg in presence of 26.3 mg C L^−1^ EPS at different time points obtained by the AF4-DAD-FLD-ICP-MS. the signals originate from different detectors: Ag^107^ from ICP-MS, DAD represents absorbance at 394 nm for nAg SPR and FLD is the fluorescence for protein tryptophan-like fluorophores. The red circle represents the formation of new peak (or bump) in protein signal coeluting with of the nAg.

To address the dynamics of eco-corona formation, AF4-DAD-FLD-ICP-MS analysis was performed over time on a suspension of 4 mg L^−1^ nAg incubated with 26.3 mg C L^−1^ EPS (Fig. 7). In the absence of EPS, the elution of nAg or their aggregates was not possible under the used conditions due to fouling of the separation membrane. Indeed, the loss of uncoated or citrate-coated nAg by fouling the membrane is one of the main factors affecting the recovery in AF4 when ionic strength of the eluent is high.^62,63^ In the presence of the EPS, nAg eluted with a retention time of 5 min based on elemental silver and characteristic nAg SPR signals (Ag^107^ and SPR at 399 nm, Fig. 7). Relatively good recoveries of (77.0 ± 8.4) % for DAD and (75.88 ± 5.4) % for Ag^107^ for ICP-MS were obtained. The enhancement of nAg elution by AF4 has been previously observed in the presence of individual proteins, such as ceruloplasmin or catalase, which form strong complexes with 20 nm nAg.^46,64^ This phenomenon was also reported for other proteins, such as superoxide dismutase, where the interaction with nAg was weaker.^46^ In this latter case, it has been suggested that proteins limit the fouling of the membrane by nAg through a screening phenomenon rather than protein–nAg complex formation. While challenging to conclusively demonstrate corona formation through the elution behavior of nAg, the presence of a distinct peak in the protein fractogram coinciding with the elution peaks of nAg was observed. This was noted in addition to the two protein peaks initially present in the EPS (Fig. S2†). These findings closely align with the results obtained by TEM (Fig. 4) and the rapid and increasing with the time adsorption of proteins (Fig. 5). Furthermore, following 24 h of incubation, there is appearance of silver species with longer retention times, and their proportion increased after 72 h. Subsequently, a concomitant decline in initial main nAg peaks is observed. This outcome is in line with the observed increase in hydrodynamic size measured by batch DLS (Fig. 2, Table S6†). Additionally, a non-negligeable proportion of proteins is detectable towards the end of the elution (*t*_ret_ > 18 min), a trend not followed by elemental silver. This suggests that proteins within the suspension may have undergone aggregation over prolongated incubation times, without involving nAg complexation.

## 4. Conclusions

A combination of multiple and complementary state-of-art techniques was employed to explore the interactions of the EPS released by diatom *C. meneghiniana* and citrate-coated nAg. The specific accent was on the study of the effect of the EPS on the nAg colloidal stability in freshwater setting, formation of EPS–nAg complex and eco-corona. The results demonstrated that EPS from *C. meneghiniana* significantly affect the fate of nAg, revealing an increased colloidal stability of citrate-coated nAg, due to the adsorption of the EPS onto the nAg surface and formation of EPS–nAg complex. The eco-corona increased stability of nAg suspended in synthetic freshwater by steric repulsion and hindering the aggregation. Moreover, adsorbed EPS played a role of bridging agent between nAg, promoting hetero-aggregation. Both main components of EPS, polysaccharides, and proteins, were found to play a role in the eco-corona formation. Fluorescence quenching study revealed that complex formation between EPS proteins and nAg occurred by hydrophobic interactions. This finding was confirmed by AF4-DAD-FLD-ICP-MS results where a formation of a new peak in protein profile in correspondence of nAg elution was observed. The dissolution of nAg was limited by EPS thus reducing the bioavailability of ionic silver, the main responsible of nAg toxicity. More broadly, these findings suggest that diatoms, by releasing EPS, can play an important role in shaping the fate and potentially the bioavailability of nAg in aquatic environments rich in the EPS.

## Data availability

Data for this article, including UV-vis, fluorescence, zeta potential and hydrodynamic size, dissolution, adsorption of polysaccharides and proteins, AF4-DAD-FLD-ICP-MS are available at YARETA at https://doi.org/10.26037/yareta:crfwhqvaxjb43p7v367ktjov3a.

## Author contributions

Rocco Gasco: conceptualization, data curation, data interpretation, investigation, methodology, formal analysis, writing – original draft. Isabelle A. M. Worms: methodology, data interpretation, investigation, writing – review & editing. Arin Kantarciyan: methodology, investigation, writing – review & editing. Vera I. Slaveykova: conceptualization, validation, data interpretation, supervision, funding acquisition, writing – review & editing.

## Conflicts of interest

The authors declare that they have no known competing financial interests or personal relationships that could have appeared to influence the work reported in this paper.

## Supplementary Material

EN-011-D4EN00232F-s001
