# Supplementary material for: Diatom-derived extracellular polymeric substances form eco-corona and enhance stability of silver nanoparticles
Source: Environ Sci Nano. 2024 Jun 25;11(10):4138–50. doi: 10.1039/d4en00232f (PMC11467845; doi:10.1039/d4en00232f)
Supplement: EN-011-D4EN00232F-s001 [file EN-011-D4EN00232F-s001.pdf]

Electronic Supplementary Material (ESI) for Environmental Science: Nano.
This journal is © The Royal Society of Chemistry 2024

Supplementary information

Diatom-Derived Extracellular Polymeric Substances Form Eco-corona and Enhance Stability of Silver Nanoparticles

Rocco Gasco*, Isabelle A.M. Worms, Arin Kantarciyan, Vera I. Slaveykova*

University of Geneva, Faculty of Sciences, Department F.-A. Forel for Environmental and Aquatic Sciences, Environemntal Biogeochemistry and Ecotoxicology, Bvd Carl-Vogt 66, 1211-Geneva Switzerland

Table of Contents

1. Culture preparation, EPS extraction and characterization 3

2. Interactions between 20 nm nAg and EPS 4

3. Transmission electron microscopy 4

4. Determination of nAg dissolution 5

5. AF4-DAD-FL-ICP-MS 5

6. Fluorescence quenching experiments 6

Table S1. Values of hydrodynamic diameter (Z-Ave), polydispersity index (PdI), Zeta potential and diameter based on size distribution with TEM of stock suspension of citrate-coated nAg. 8

Table S2. Compositions of theSynthetic Freshwater Medium (SFM) + Si used for diatom growth and simplified SFM used for the nAg-EPS incubation experiments. 9

Table S3. Operating conditions for fractionation and detection of nAg by AF4-MD-ICP-MS. 10

Table S4. Composition of EPS and various concentration of EPS tested 11

Table S5. Band assignments for the peaks collected in the FTIR spectrum of the EPS. 12

Table S6. Time evolution of the hydrodynamic diameter (Z-Ave), polydispersity index (PdI), Zeta potential and diameter based on size distribution with TEM of stock suspension of citrate-coated nAg in the absence and presence of different EPS concentrations. 13

Figure S1. Spectra collected to characterize the EPS. 14

Figure S2. Fractograms obtained by AF4-FLD illustrating the t 15

Figure S3. SPR-UV-vis absorption spectra of 4 mg L^{-1} nAg in absence and presence of different concentrations of EPS 16

Figure S4. TEM images of aggregates formation from 4 mg L^{-1} nAg. 17

Figure S5. TEM images of EPS amorphous layer formed on 4 mg L^{-1} nAg after 24h. 18

Figure S6. Concentrations of EPS components involved in corona formation 19

Figure S7. Fluorescence quenching results at different temperatures. 20

1. Culture preparation, EPS extraction and characterization

Axenic culture of unicellular diatom *Cyclotella meneghiniana* (strain CCAC 0039) was obtained from Central Collection of Algal Cultures, University of Duisburg-Essen, Germany. The diatoms were grown under axenic conditions, in a specialized incubator at 20±1 °C, fluorescent daylight (90 µmol $s^{-1}m^{-2}$) in a 16:8 light/dark cycle photoperiod and a constant agitation of 60 rpm. The culture medium used was Synthetic Freshwater Medium (SFM)) with the addition of silica content (SFM+Si) specific for the growth of diatoms (**Table S2**) [1]. The dissolved EPS were harvested at the stationary growth phase of diatoms, corresponding to a cell density of $1.2x10^6$ cells mL^{-1} by removing the algae using centrifugation at 4121×*g* for 5 minutes (Multifuge X4 and X4R Pro Centrifuges, Thermo Fisher, US). The supernatant containing the exudates was collected and filtered on 0.45 µm filter (VWR® filter, PES membrane, sterile) to remove eventual remaining cells. Afterward, the filtrate was pull in ultrafiltration centrifugal filter devices (Macrosep™ Advance centrifugal devices) with a 1 kDa cut-off ultrafiltration membrane (Omega™) and centrifuged at 4121×*g* and 4 °C with centrifuge (Multifuge X4 and X4R Pro Centrifuges, Thermo Fisher, US). This step allows to isolate EPS > 1 kDa and concentrate them by a factor of 50 (v/v).

A combination of different analytical tools was employed to characterize the diatom derived EPS. The presence of fluorophore groups in the EPS was probed by collecting three-dimensional excitation emission matrix (3D-EEM) fluorescence spectra using a fluorescence spectrofluorometer (Cary Eclipse, Agilent, USA). The scans were performed within the excitation range of 200 to 450 nm and the emission range of 200 to 500 nm, using the SFM medium as a blank. Fourier transform infrared (FTIR) spectra of freeze-dried EPS was collected in absorbance mode spanning a range of wavenumber from 500 to 4000 cm^{-1} using an ATR spectrophotometer (FTIT spectrum two+ ATR, Perkin Elmer, USA) to explore the presence of different functional groups.

2. Interactions between 20 nm nAg and EPS

To study the interactions of EPS and citrate-coated silver nanoparticles (nAg), different concentrations of EPS (10.5, 26.3, 52.5 and 105.0 mg C L^{-1}) were mixed with 4 mg Ag L^{-1} of 20 nm - sized nAg. To this end the preconcentrated EPS released were diluted into simplified SFM medium maintaining the same ionic strength to avoid the conformation changes, unfolding of proteins and aggregation due to changes in ionic strength [2, 3]. The changes in the size distribution, surface properties and stability of nAg suspensions were studied at 2, 24, 48 and 72 h.

3. Transmission electron microscopy

nAg size distribution was characterized by transmission electron microscopy (TEM, Talos™ L120C TEM, Thermo Fisher, USA) at 120 kV at the incubation times of 2, 24, and 72h in the absence and presence of 10.5 or 105 mg C L^{-1} EPS. Precisely, 5 µL of sample were deposited onto ultrathin carbon-coated copper grids (Carbon Film 400 Mesh, Copper, Electron Microscopy Sciences, USA) negatively charged for 1 min and then paper dried to avoid as possible artifact from the drying process. Negative staining with uranyl acetate was used to study the ecocorona formation and the organic matter in the EPS. After sample deposition onto the grid, it was placed into a 40 µL 2% uranyl acetate drop for 2 sec and consecutively into another 20 µL drop for 30 sec before the paper drying step. The size distribution of the nAg was evaluated on TEM images counting at least 120 particles for each condition using Fiji software [4].

4. Determination of nAg dissolution

The influence of EPS on dissolution of nAg was obtained by centrifugation using a procedure reported in the literature[5] after adaptation. Shortly, the suspensions containing nAg and increasing concentrations of EPS were centrifuged for 3h at 24 000×*g* at 4 °C (5417R Refrigerated Centrifuge, Eppendorf, USA). The supernatant, containing the dissolved Ag ions was collected and acidified with HNO_3 (Merck, Germany) to a final concentration of 25% HNO_3 (v/v), then placed at 50 °C overnight to digest the organic matter residues. The digested samples were diluted to a final concentration of 2% HNO_3 (v/v) and silver content was measured by ICP-MS (7700x Q-ICP-MS Agilent, USA). The dissolved silver ions content was evaluated after 3, 24 and 72h of incubation. The results are expressed as a percentage of dissolved silver ions in comparison to the initial concentration of nAg. All experiments were performed in triplicate.

5. AF4-DAD-FL-ICP-MS

Asymmetrical flow field-flow fractionation (AF4, AF2000 Focus) coupled with diode array (DAD) and fluorescence (FL) detectors (Postnova Analytics, Germany) and ICPMS (7700x Q-ICP-MS Agilent, USA) was employed to obtain multiple information about the EPS. The AF4-DAD-FL-ICP-MS was used to study the colloidal stability of the nAg in presence of EPS. The absorbance signal was recorded at SPR absorbance peak of nAg (394nm) with the DAD and Ag associated with EPS was measured by ICP-MS. The optimized parameters for sample fractionation as well as different detectors are provided in **Table S3**. To determine the molecular weight of the protein fraction of the EPS, four different standard proteins of different molecular weights were used to derive an external calibration curve: equine myoglobin (17 kDa), bovine hemoglobin (36 kDa), horseradish peroxidase (40 kDa) and BSA (66 kDa).

6. Fluorescence quenching experiments

The interaction between nAg and protein fraction of the EPS was investigated studying the quenching effect of nAg (Q) on the tryptophan-like groups of proteins in EPS. The samples were excited at 280 nm and the emission spectrum was acquired in the range 295 to 450 nm. Tryptophan-like fluorophores are characterized by two peaks (A: Ex/Em: 280/348 nm and B: Ex/Em: 255/333 nm). The peak B was excluded from quenching experiments due to interferences with emission of the nAg.

Fluorescence spectrometry measurements were performed on a Fluorescence Spectrometer (Agilent Cary Eclipse, USA) using a 1 cm quartz cuvette. Both excited and emission slit widths were set to 10.0 nm and the scanning speed was 1200 nm min^{-1}.

The effect of temperature on the EPS binding to nAg was studied at absolute temperatures of 298, 303 and 308 K. The fixed EPS concentration of 26.3 mg C L^{-1} (equivalent to 2.1 mg BSA L^{-1}) was incubated with increasing concentration (0.1-4 mg L^{-1}) of nAg for 30 min. The results relative to quenching process were used to evaluate quenching mechanism of nAg with EPS using the Stern-Volmer equation (Eq. (1)) [7] .

$$F_0/F = 1 + k_q \tau_0[\mathrm{Q}] = 1 + K_{SV}[\mathrm{Q}] \qquad (1)$$

where F_0 and F are the fluorescence intensities of protein fraction of the EPS in absence and presence of the nAg, k_q is the biomolecular quenching rate constant, K_{SV} is the Stern-Volmer constant, τ_0 is the average lifetime of biomolecule (10^{-8} s) in the absence of the nAg, and [Q] represent the concentration of nAg. The slope of the linear regression of the Stern-Volmer equation (Eq. (1)) corresponds to the K_{SV} which was used to calculate k_q.

The binding constant K_b and the number of binding sites n for static quenching processes was determined by the linear regression of the following equation (Eq. (2))[7] .

$$\log[(F_0\text{-}F)/F] = \log K_\mathrm{b} + n\log[\mathrm{Q}] \qquad (2)$$

The values of enthalpy change (ΔH°) and entropy change (ΔS°) were determined using the linear regression of Van't Hoff equation (Eq. (3)), where R is the gas constant. After it was possible to evaluate (ΔG°) with Gibbs-Helmholtz equation (Eq. (4)).

$$\ln K_b = -\Delta H^\circ /RT + \Delta S^\circ /R \qquad (3)$$

$$\Delta G^\circ = \Delta H^\circ - T\Delta S^\circ \qquad (4)$$

To see the protein corona formation and the possible saturation point, fixed concentration of 2 mg L^{-1} nAg was incubated with EPS at different concentration of 6.6, 10.5, 26.3, 52.5, 105.0 mg C L^{-1} corresponding to 0.53-8.40 mg BSA L^{-1} equivalent for 30 min at room temperature, followed by fluorescence measurements to assess percent of quenching effect (% $F\ F_0^{-1}$).

Table S1. Values of hydrodynamic diameter (Z-Ave), polydispersity index (PdI), Zeta potential and diameter based on size distribution with TEM of stock suspension of citrate-coated nAg.

Parameter	Starting conditions
**Z-Ave (d. nm)**	32.93 ± 0.14
**PdI**	0.265 ± .001
**Zeta potential (mV)**	-39.93 ± 1.62
**TEM diameter (nm)**	19.9 ± 2.8 (Nanocomposix value)

Table S2. Compositions of theSynthetic Freshwater Medium (SFM) + Si used for diatom growth and simplified SFM used for the nAg-EPS incubation experiments.

Compound	Final concentration
HEPES (1.00 mM)	1.00 mM
$Ca(NO_3)_2$ x $4H_2O$	0.21 mM
$MgSO_4$ x $7H_2O$	0.20 mM
K_2HPO_4x $3H_2O$	13.20 µM
$NaNO_3$	0.35 mM
Na_2CO_3	0.19 mM
H_3BO_3	16.00 µM
Na_2SiO_3x $9H_2O$	0.50 mM
Vitamins*	
Vitamin B12	0.15 nM
Biotin	4.10 nM
Thiamine-HCl	0.30 µM
Niacinamide	0.80 nM
Trace metals solution*	
Na_2EDTA x 2 H_2O	11.71 µM
$FeCl_3$ x 6 H_2O	11.65 µM
K_2CrO_4	9.99 nM
$CoCl_2$ x $6H_2O$	42.03 nM
$CuSO_4$ x $5H_2O$	10.01 nM
$MnCl_2$ x $4H_2O$	0.90 µM
Na_2MoO_4x $2H_2O$	78.12 nM
$NiSO_4$x $6H_2O$	10.27 nM
H_2SeO_3	10.08 nM
Na_3VO_4	10.00 nM
$ZnSO_4$ x $7H_2O$	76.51 nM

*These components of the growth medium were removed in the simplified SFM medium

Table S3. Operating conditions for fractionation and detection of nAg by AF4-MD-ICP-MS.

**Fractionation parameters**		
Spacer	350 µm	
Membrane	10 kDa (Regenerate cellulose, Postnova)	
Detector flow	0.7 mL min^{-1}	
Run time	40 min	
Eluent	10 mM HEPES pH 7	
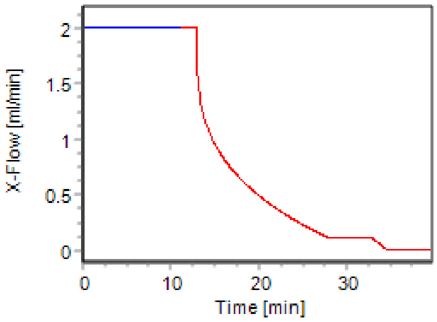	*Focus step*	InjF=0.2 mL min^{-1}<br>Cte XF=2 mL min^{-1}<br>FPF=2.5 mL min^{-1}<br>Injection time: 10 min<br>Transition time: 1 min
	Elution	Cte XF=2 mL min^{-1}; 2 min<br>Grad XF=2-0.1 mL min^{-1}15 min<br>Cte XF=0.1 mL min^{-1}; 5 min<br>Total elution time: 22 min
	Release step	XF=0 mL min^{-1}; 5 min
**Detection**		
Diode Array Detector (DAD)	Wavelengths: 280 nm (Abs Protein); 394 nm (SPR nAgs)	
Fluorescence Detector (FLD)	Ex/Em: 280/350 nm (tryptophan-like fluorophores)	
ICP-MS isotopes	^{107}Ag, ^{109}Ag	

XF = cross flow rate

FPF = Focus pump flow

Grad = gradient power decay exponent 0.3

InjF = Injection flow

Table S4. Composition of EPS and various concentration of EPS tested. For TOC and TN, values of the growth medium were subtracted.

Property	Concentrated EPS	105.0 mg C L^{-1} EPS	52.5 mg C L^{-1} EPS	26.3 mg C L^{-1} EPS	10.5 mg C L^{-1} EPS
Protein content (mg L^{-1} BSA eq.)	43.06 ± 6.27 (RSD%: 14.55)	8.6	4.3	2.15	0.86
Carbohydrate content (mg L^{-1} glucose eq.)	610.07 ± 31.75 (RSD%: 5.20)	122	61	30.5	12.2
Total Organic Carbon (mg C L^{-1})	525.25 ± 29.37 (RSD%: 5.59)	105.0	52.5	26.25	10.5
Total dissolved nitrogen (mg N L^{-1})	99.87 ± 1.77 (RSD%: 2.38)	5	2.5	1.25	0.5
Zeta Potential in Expo Med (mV)	-13.75 ± 1.54 (RSD%: 11.22)	---	---	---	---

Table S5. Band assignments for the peaks collected in the FTIR spectrum of the EPS.

Wavenumber (cm^{-1})	Band assignment	Macromolecules	Reference
827	NH_2 wag in primary amines of proteins	Proteins	[8]
935	P-O-C antisym stretch in organophosphorus compounds	Phospholipids	[8, 9]
1039	C-O- and C-O-C stretch carbohydrates	Polysaccharides	[8]
1077	C-O stretch carbohydrates	Polysaccharides	[8]
1388	C-H bending of aldehydes	Polysaccharides	[10]
1455	O-H bending in carboxylic acids	Polysaccharides	[8]
1527	Amide absorption<br>Aromatic C=C	Proteins	[11]
1648	N-H primary amines bending in protein and stretching amide	Proteins	[11]
1730	C=O stretch in ester in lipids/phospholipids	Lipids/phospholipids	[8]
2950-2838	C-H antisym and sym stretch of fatty acids	Lipids	[8]
3340	NH_2 sym/antisym stretch in primary amines of proteins<br>O-H stretch of carbohydrates	Proteins<br>Polysaccharides	[8]

Table S6. Time evolution of the hydrodynamic diameter (Z-Ave), polydispersity index (PdI), Zeta potential and diameter based on size distribution with TEM of stock suspension of citrate-coated nAg in the absence and presence of different EPS concentrations.

		ZetaSizer measurements				
		Time points				
	**Sample**	**2min**	**1h**	**2h**	**24h**	**72h**
**Z-Ave (d. nm)**	*0 mg C L^{-1}*	30.38±2.23	51.59±0.63	68.77±0.68	231.71±8.18	364.64±18.88
	10.5 mg C L^{-1}	32.31±2.34	37.91±1.70	38.50±0.91	46.20±0.39	54.36±1.04
	26.3 mg C L^{-1}	38.01±0.93	38.82±0.96	41.21±0.83	50.00±0.37	61.27±1.29
	52.5 mg C L^{-1}	40.51±0.20	42.50±0.45	44.60±1.54	53.36±1.93	68.99±1.65
	105.0 mg C L^{-1}	42.85±2.36	45.97±2.05	46.49±2.45	56.90±0.24	75.57±1.20
**Polydispersity (PdI)**	*0 mg C L^{-1}*	0.255±0.059	0.267±0.002	0.259±0.004	0.334±0.011	0.509±0.054
	10.5 mg C L^{-1}	0.260±0.144	0.251±0.049	0.268±0.034	0.256±0.008	0.251±0.011
	26.3 mg C L^{-1}	0.232±0.047	0.282±0.003	0.267±0.026	0.256±0.007	0.242±0.009
	52.5 mg C L^{-1}	0.279±0.032	0.279±0.004	0.294±0.013	0.249±0.007	0.247±0.009
	105.0 mg C L^{-1}	0.374±0.039	0.269±0.035	0.284±0.039	0.258±0.005	0.249±0.009
**Zeta potential (mV)**	*0 mg C L^{-1}*	---	-23.07±1.62	-24.30±0.44	-24.42±0.66	-25.23±0.38
	10.5 mg C L^{-1}	---	-23.18±1.15	-22.86±1.30	-22.67±0.99	-19.30±1.32
	26.3 mg C L^{-1}	---	-22.72±0.89	-22.83±0.90	-20.05±0.79	-21.39±0.96
	52.5 mg C L^{-1}	---	-21.43±0.66	-21.62±0.91	-19.12±0.60	-21.40±0.78
	105.0 mg C L^{-1}	---	-22.2±0.94	-23.06±1.52	-21.75±1.02	-21.4±1.00
		TEM measurements				
**Size (nm)**	*0 mg C L^{-1}*	---	---	20.77±4.56	22.14±7.41	22.32±6.33
	10.5 mg C L^{-1}	---	---	19.30±3.24	19.83±4.40	19.44±3.11
	105.0 mg C L^{-1}	---	---	19.52±4.24	19.52±4.24	19.21±4.36

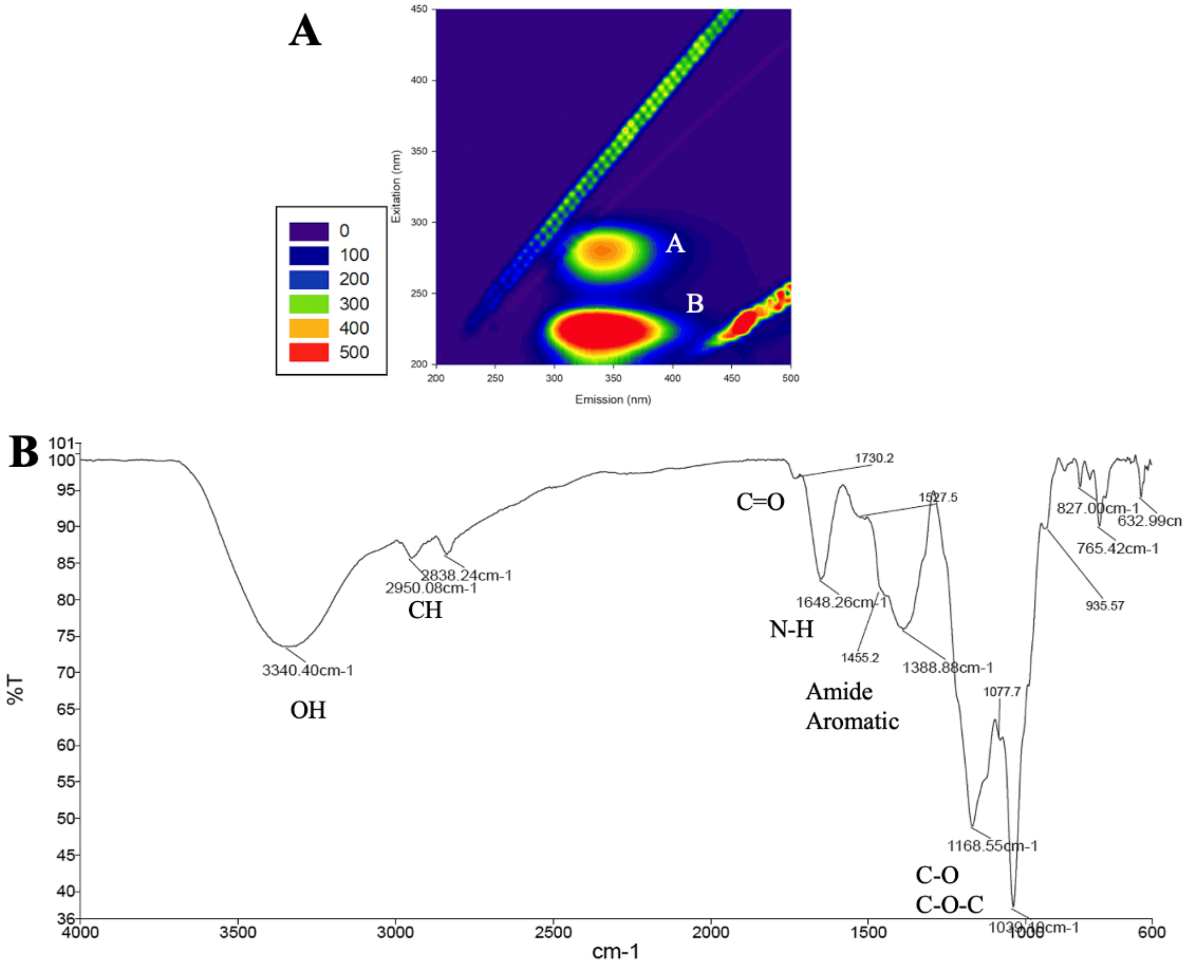

Figure S1. Spectra collected to characterize the EPS. (A) 3D-EEM spectrum of 52.5 mg C L^{-1} EPS, 1: Tryptophan-like peak (**Peak** A Ex/Em: 282/333 and **Peak** B Ex/Em: 230/308 nm) related to protein fluorescence. (B) FTIR spectrum of the freeze-dried EPS.

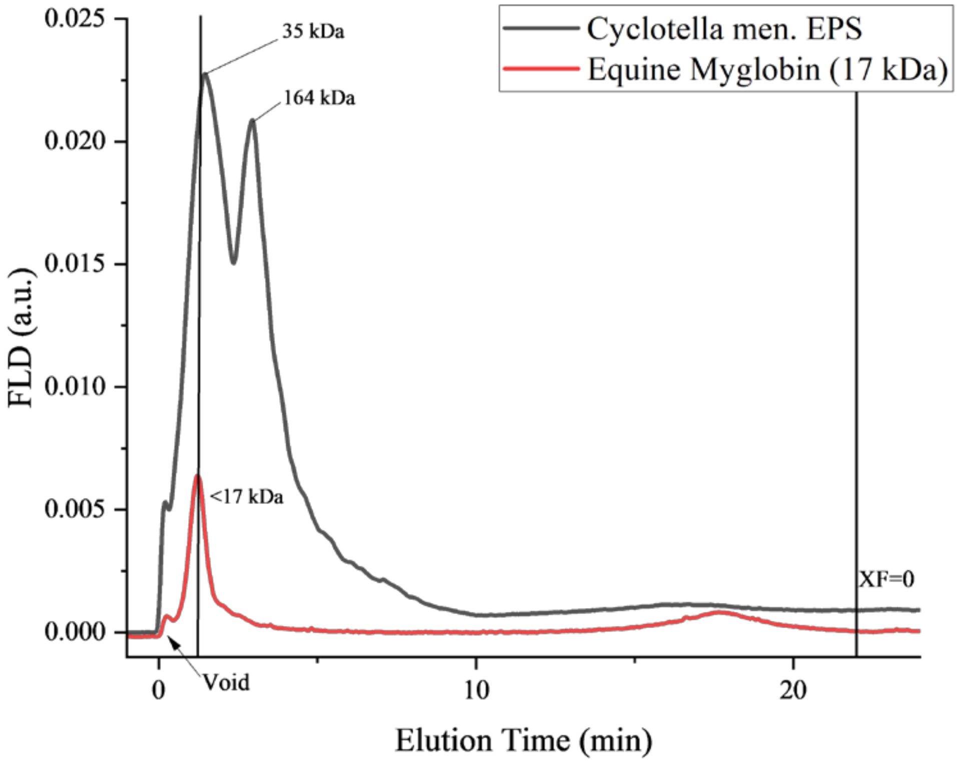

Figure S2. Fractograms obtained by AF4-FLD illustrating the tryptophan-like fluorophores in the EPS (black) and equine myoglobin (17 kDa, red). Injected volume, Vinj: 500 µL, Concentrations of the EPS: f 26.3 mg C L^{-1} EPS. Equine myoglobin standard is used for MW evaluation of the protein fraction in EPS.

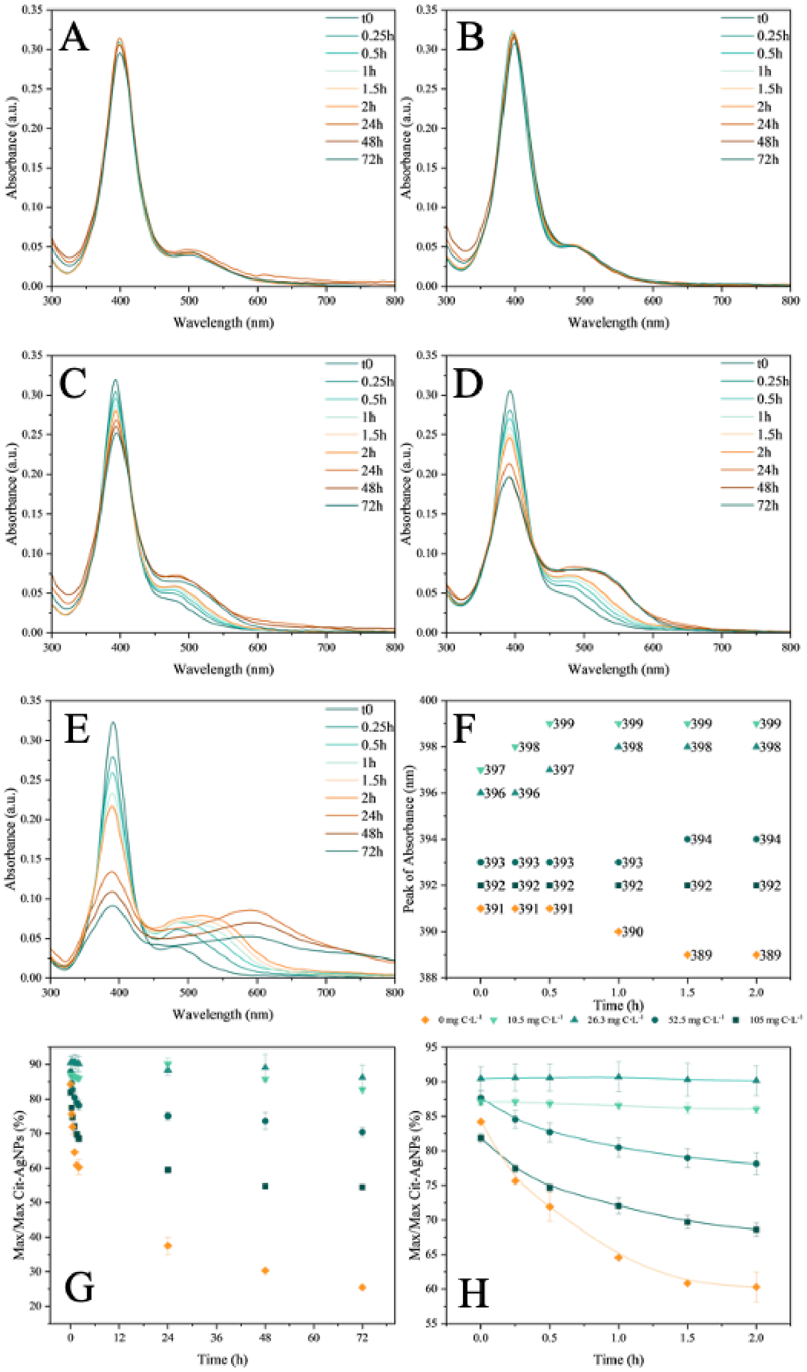

Figure S3. SPR-UV-vis absorption spectra of 4 mg L^{-1} nAg in absence and presence of different concentrations of EPS**.** SPR-UV-Vis of nAg dispersed in simplified SFM medium in the presence of (A) 10.5 mg C/L EPS, (B) 26.3 mg C/L EPS, (C) 52.5 mg C/L EPS, (D) 105.0 mg C/L EPS and (E) 0 mg L^{-1} EPS at different reaction times. (F) Values of the peak of absorbance collected during the short-term incubation (0-2h). (G) and (H) showed the corresponding changes of the ratio between the absorbance peak at the reaction time and initially (Max/Max Cit-AgNPs) in absence and presence of different EPS concentrations (0, 10.5, 26.3, 52.5, 105.0 mg C L^{-1}). (G) Long-term trends of Max/Max Cit-AgNPs (0-2h, 24, 48 and 72h) and (H) short-term trends (from 0 to 2h).

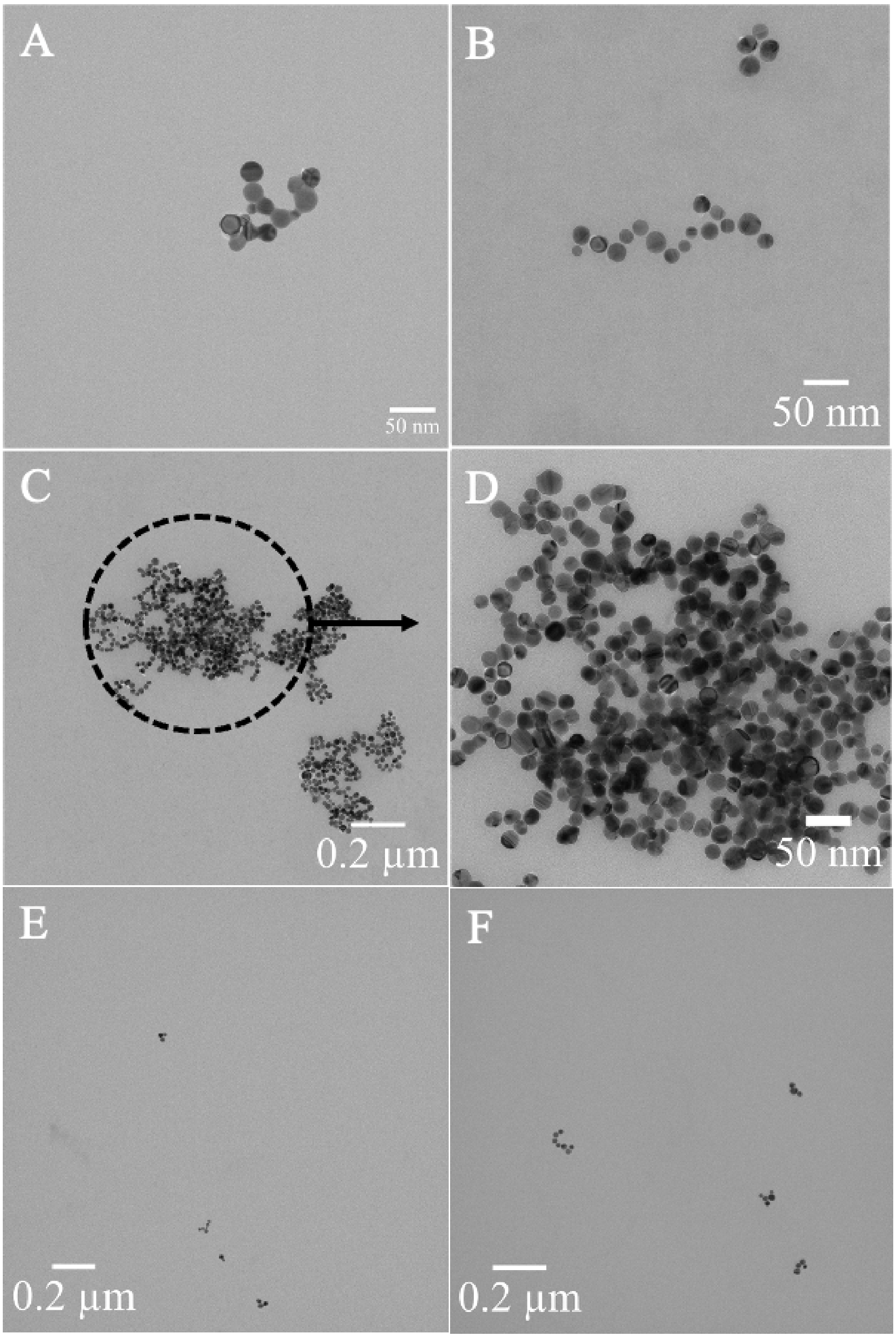

Figure S4. TEM images of aggregates formation from 4 mg L^{-1} nAg. (A) Ostwald ripening phenomenon in absence of EPS (0 mg C L^{-1}) after 2h. (B) Presence of EPS (105 mg C L^{-1}) the nAg are more separated due to EPS adsorbed and consequent steric repulsion after 2h. (C) and (D) big homo aggregates of nAg in absence of EPS after 24h. (E) and (F) smaller hetero aggregates due to presence of 10.5 and 105.0 mg C L^{-1} EPS, respectively, after 24h.

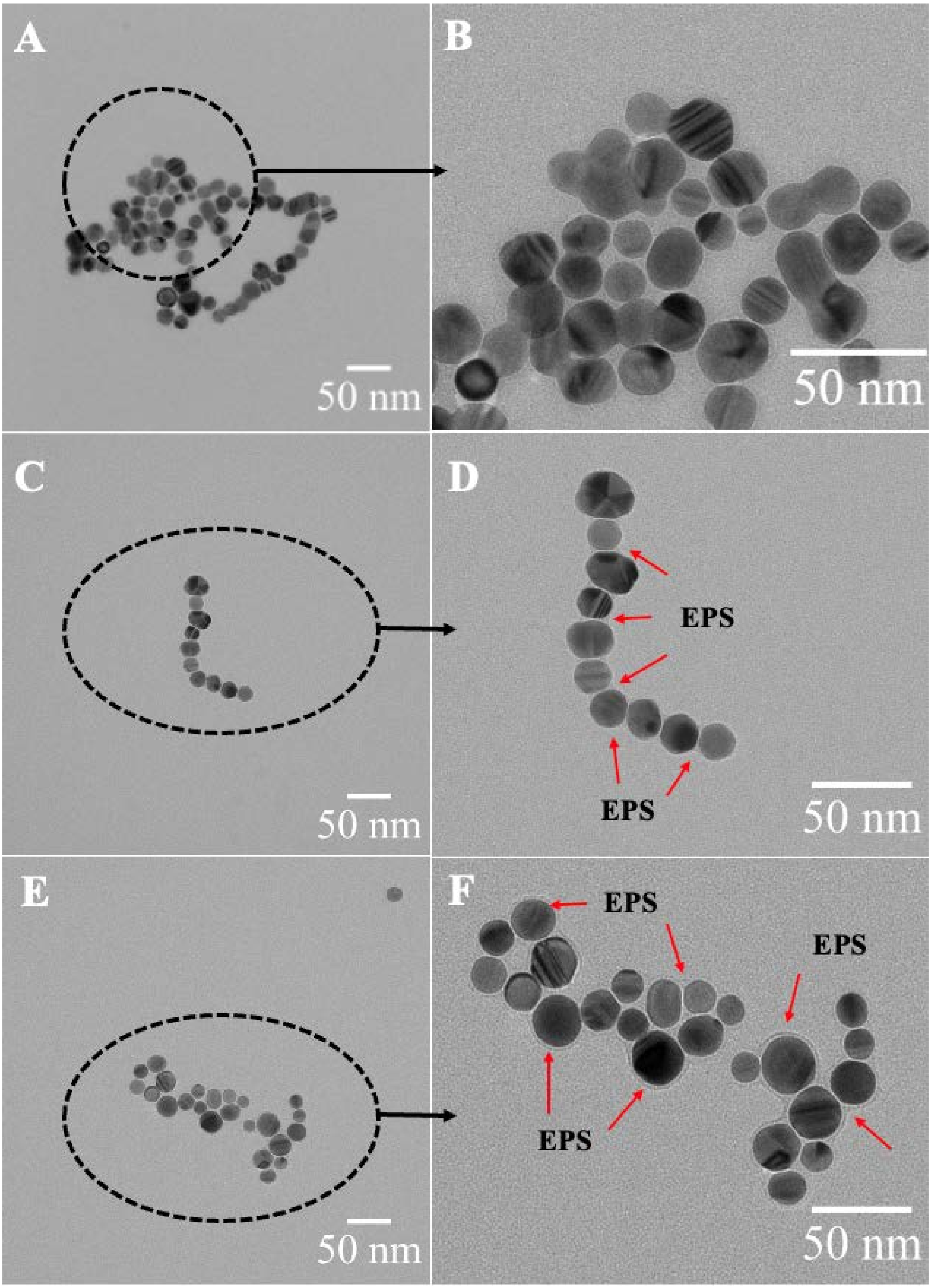

Figure S5. TEM images of EPS amorphous layer formed on 4 mg L-1 nAg after 24h incubation with EPS. The nAg images were collected in presence of 0 mg C L-1 EPS (A) and (B), 10.5 mg C L-1 EPS (C) and (D) and 105 mg C L-1 EPS (E) and (F) after 24h incubation.

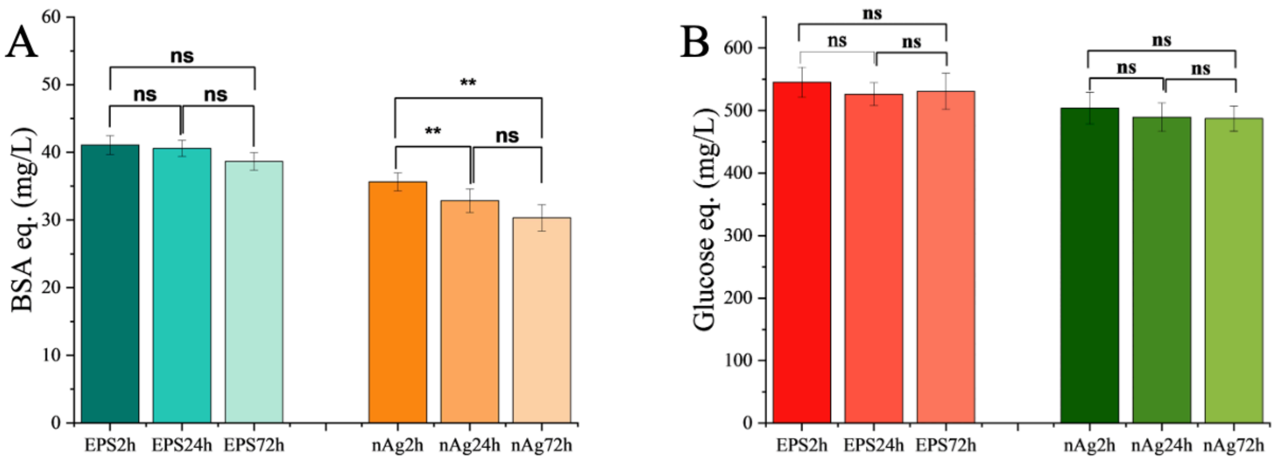

Figure S6. Concentrations of EPS components involved in corona formation**.** F-test results (ns: p>0.05, *: p≤0.05; **: p≤0.01; ***: p≤0,001; **** p≤0,0001). EPSxh: time related components (proteins/polysaccharides) concentration in EPS without nAg. nAgxh: time related components (proteins/polysaccharides) concentration in EPS incubated with nAg. The statistical treatment showed no differences in the concentrations of proteins (A) and polysaccharides (B) due to incubation conditions, meaning that the changing in concentrations are due to adsorption of EPS components.

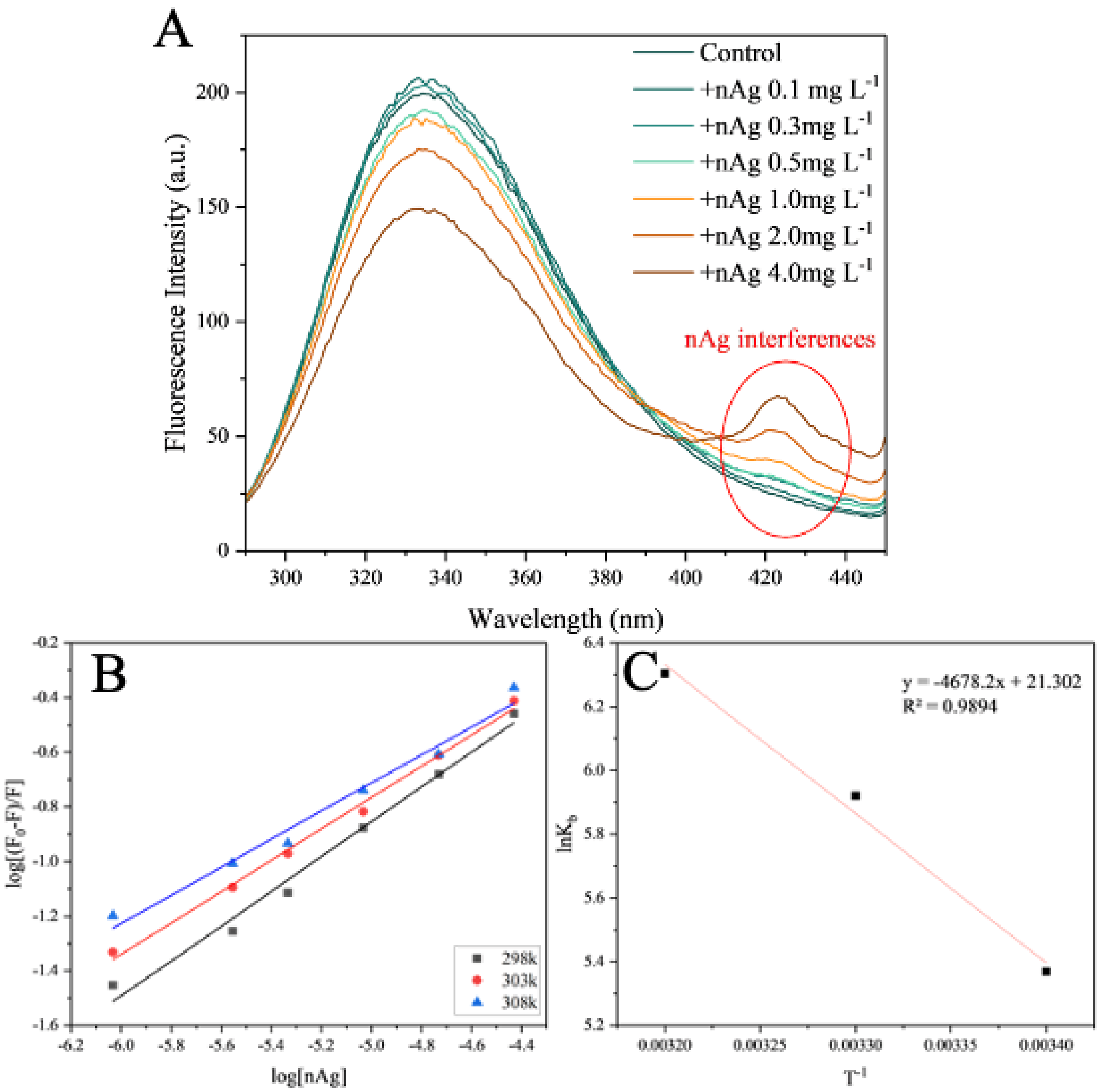

Figure S7. Fluorescence quenching results at different temperatures. (A) Interference fromnAg in fluorescence intensities of tryptophan-like fluorophore peak (Ex: 250 nm, Em: 290-450 nm) of constant 26.3 mg C L^{-1} EPS protein in presence of increasing nAg concentrations (0.1, 0.3, 0.5, 1.0, 2.0, 4 mg L^{-1}) with the Stern-Volmer plot at 298 K derived for the binding of nAg to EPS. (B) Double logarithmic plots for the quenching results at different temperatures of constant EPS (26.3 mg C L^{-1} or 2.1 BSA mg L^{-1} eq.) with variable nAg concentrations (0.3-4 mg L^{-1}). (C) Van't Hoff plots: ln of quenching rate constant of the EPS tryptophan-like fluorescence versus inverse of temperature.

References

1. J. P. Santos, W. Li, A. A. Keller and V. I. Slaveykova, Mercury species induce metabolic reprogramming in freshwater diatom *Cyclotella meneghiniana*, *J Hazard Mater*, 2024, **465**, 133245.
2. W.-q. Wang, J.-j. Li, J.-y. Zhou, M.-x. Song, J.-c. Wang, X. Li, C.-C. Tang, M.-l. Lu and R.-x. Gu, The effect of ion environment changes on retention protein behavior during whey ultrafiltration process, *Food Chemistry: X*, 2022, **15**, 100393.
3. E. Sahin, A. O. Grillo, M. D. Perkins and C. J. Roberts, Comparative Effects of pH and Ionic Strength on Protein–Protein Interactions, Unfolding, and Aggregation for IgG1 Antibodies, *Journal of Pharmaceutical Sciences*, 2010, **99**, 4830-4848.
4. J. Schindelin, I. Arganda-Carreras, E. Frise, V. Kaynig, M. Longair, T. Pietzsch, S. Preibisch, C. Rueden, S. Saalfeld, B. Schmid, J. Y. Tinevez, D. J. White, V. Hartenstein, K. Eliceiri, P. Tomancak and A. Cardona, Fiji: an open-source platform for biological-image analysis, *Nat Methods*, 2012, **9**, 676-682.
5. I. Fernando and Y. Zhou, Impact of pH on the stability, dissolution and aggregation kinetics of silver nanoparticles, *Chemosphere*, 2019, **216**, 297-305.
6. I. Fernando, D. Lu and Y. Zhou, Interactive influence of extracellular polymeric substances (EPS) and electrolytes on the colloidal stability of silver nanoparticles, *Environmental Science: Nano*, 2020, **7**, 186-197.
7. M. Waghmare, B. Khade, P. Chaudhari and P. Dongre, Multiple layer formation of bovine serum albumin on silver nanoparticles revealed by dynamic light scattering and spectroscopic techniques, *Journal of Nanoparticle Research*, 2018, **20**.
8. A. S. Adeleye and A. A. Keller, Interactions between Algal Extracellular Polymeric Substances and Commercial TiO_2 Nanoparticles in Aqueous Media, *Environ Sci Technol*, 2016, **50**, 12258-12265.
9. P. Di Martino, Extracellular polymeric substances, a key element in understanding biofilm phenotype, *AIMS Microbiol*, 2018, **4**, 274-288.
10. K. Zhou, Y. Hu, L. Zhang, K. Yang and D. Lin, The role of exopolymeric substances in the bioaccumulation and toxicity of Ag nanoparticles to algae, *Sci Rep*, 2016, **6**, 32998.
11. Y. Yang, S. Zheng, R. Li, X. Chen, K. Wang, B. Sun, Y. Zhang and L. Zhu, New insights into the facilitated dissolution and sulfidation of silver nanoparticles under simulated sunlight irradiation in aquatic environments by extracellular polymeric substances, *Environmental Science: Nano*, 2021, **8**, 748-757.
